# A single-cross, RNA interference-based genetic tool for examining the long-term maintenance of homeostatic plasticity

**DOI:** 10.3389/fncel.2015.00107

**Published:** 2015-03-26

**Authors:** Douglas J. Brusich, Ashlyn M. Spring, C. Andrew Frank

**Affiliations:** ^1^Department of Anatomy and Cell Biology, Carver College of Medicine, University of IowaIowa City, IA, USA; ^2^Interdisciplinary Graduate Program in Genetics, University of IowaIowa City, IA, USA; ^3^Interdisciplinary Programs in Genetics, Neuroscience, and MCB, University of IowaIowa City, IA, USA

**Keywords:** homeostatic plasticity, *Drosophila melanogaster*, neuromuscular junction, RNAi screening, synaptic transmission, Ca_V_2 channels, cysteine string protein, phospholipase C beta

## Abstract

Homeostatic synaptic plasticity (HSP) helps neurons and synapses maintain physiologically appropriate levels of output. The fruit fly *Drosophila melanogaster* larval neuromuscular junction (NMJ) is a valuable model for studying HSP. Here we introduce a genetic tool that allows fruit fly researchers to examine the lifelong maintenance of HSP with a single cross. The tool is a fruit fly stock that combines the *GAL4/UAS* expression system with RNA interference (RNAi)-based knock down of a glutamate receptor subunit gene. With this stock, we uncover important new information about the maintenance of HSP. We address an open question about the role that presynaptic Ca_V_2-type Ca^2+^ channels play in NMJ homeostasis. Published experiments have demonstrated that hypomorphic missense mutations in the Ca_V_2 α1a subunit gene *cacophony* (*cac*) can impair homeostatic plasticity at the NMJ. Here we report that reducing *cac* expression levels by RNAi is not sufficient to impair homeostatic plasticity. The presence of wild-type channels appears to support HSP—even when total Ca_V_2 function is severely reduced. We also conduct an RNAi- and electrophysiology-based screen to identify new factors required for sustained homeostatic signaling throughout development. We uncover novel roles in HSP for Drosophila homologs of Cysteine string protein (CSP) and Phospholipase Cβ (Plc21C). We characterize those roles through follow-up genetic tests. We discuss how CSP, Plc21C, and associated factors could modulate presynaptic Ca_V_2 function, presynaptic Ca^2+^ handling, or other signaling processes crucial for sustained homeostatic regulation of NMJ function throughout development. Our findings expand the scope of signaling pathways and processes that contribute to the durable strength of the NMJ.

## Introduction

Animal nervous systems continually face challenges to normal function. When encountering neuronal stress, the outputs of synapses and circuits must be kept within a physiologically appropriate range. This restriction requires the activity of homeostatic regulatory systems. Homeostatic plasticity is a conserved principle across metazoan nervous systems. This fact is demonstrated by studies examining nematode, insect, crustacean, and mammalian synaptic preparations (Perez-Otano and Ehlers, 2005; Marder and Goaillard, 2006; Turrigiano, 2008; Pozo and Goda, 2010; Davis, 2013; Davis and Müller, 2015). Few molecules required for homeostatic synaptic plasticity (HSP) have been categorized into coherent signaling pathways. This is a gap in knowledge that limits our understanding of how neurons and synapses maintain stable function.

The *Drosophila melanogaster* third instar larval neuromuscular junction (NMJ) is a superb synapse for studying the molecular underpinnings of HSP (Frank, 2014a). At the fruit fly NMJ, genetic and pharmacological manipulations can be used to decrease the sensitivity of postsynaptic glutamate receptors to single vesicles of glutamate (decreased quantal size) (Petersen et al., 1997; Frank et al., 2006; Frank, 2014a). Decreased quantal size triggers retrograde (muscle-to-nerve) signaling that drives increased neurotransmitter release (increased quantal content, QC). As a result of this homeostatic signaling process, normal levels of muscle excitation are maintained.

Robust NMJ regulation has been exploited in genetic screens to uncover molecules required for HSP. One approach employs acute application of the glutamate receptor inhibitor, philanthotoxin-433 (PhTox) on semi-intact NMJ preparations of Drosophila larvae (Frank et al., 2006). Using Drosophila mutants, this approach has uncovered factors required for a short-term induction of synaptic homeostasis at the NMJ (10 min PhTox treatment), including presynaptic Ca_V_2-type Ca^2+^ channels (Frank et al., 2006; Müller and Davis, 2012), K_V_ potassium channels (Bergquist et al., 2010), epithelial sodium (ENaC) channels (Younger et al., 2013), BLOC-1 complex members (biogenesis of lysosome-related organelles complex-1) (Dickman and Davis, 2009), SNARE complex members (soluble N-ethylmalemide-sensitive factor attachment receptors) (Dickman et al., 2012), Rab3-GAP (Müller et al., 2011), RIM (Rab3 interacting molecule) (Müller et al., 2012), RIM binding protein (Müller et al., 2015), and secreted endostatin (Wang et al., 2014). Some of these proteins gate important presynaptic molecular events such as an increase in Ca^2+^ influx or an increase in the size of the readily releasable pool of presynaptic vesicles (Weyhersmüller et al., 2011; Müller and Davis, 2012; Müller et al., 2012; Younger et al., 2013). These presynaptic events mirror salient aspects of HSP in mammalian neurons (Murthy et al., 2001; Burrone et al., 2002; Zhao et al., 2011). Therefore, homeostatic processes at the Drosophila NMJ appear to target fundamentally conserved mechanisms that are discoverable by genetic approaches.

The aggregate research at the NMJ suggests overlapping (yet distinct) classes of molecules are required for the acute induction of HSP and the long-term maintenance of HSP (Frank, 2014a). However, acute application of PhTox misses notable factors needed for the continued expression of synaptic homeostasis throughout life, such as the Rho-type guanine exchange factor Ephexin (Frank et al., 2009), the pair-rule transcription factor Gooseberry (Marie et al., 2010), and the protein translation regulator Target of Rapamycin (TOR) (Penney et al., 2012). Alternative approaches are required to identify and elucidate signaling processes the NMJ employs to maintain faithful neurotransmission in response to chronic challenges met throughout development. Signaling processes needed for the prolonged developmental expression of synaptic homeostasis at the Drosophila NMJ could serve a similar function in higher organisms.

A null Drosophila *GluRIIA* glutamate receptor subunit mutation (Petersen et al., 1997) is valuable for characterizing molecules that work to maintain homeostatic plasticity for extended developmental time (Frank et al., 2009; Marie et al., 2010; Penney et al., 2012; Frank, 2014a). *GluRIIA* loss decreases quantal size, and the NMJ responds with a homeostatic increase in presynaptic release (Petersen et al., 1997; DiAntonio et al., 1999). Yet Drosophila *GluRIIA* mutations are not perfectly ideal for large-scale, high-throughput genetic approaches to identify homeostatic factors. Use of these mutations in screens requires generations of genetic crossing, recombination (depending upon the genomic location of the screened mutation to be tested), and the generation of homozygous double mutants. All of this work needs to be completed prior to conducting electrophysiological analyses. Partial impairment of *GluRIII* (also known as *GluRIIC*) gene function presents an alternate possibility. GluRIII is an essential glutamate receptor subunit; null *GluRIII* mutations are embryonic lethal, but *GluRIII* mutant animals can be rescued to viability with low levels of *GluRIII* gene expression (Marrus et al., 2004). Third instar larval *GluRIII* hypomorphs have decreased quantal size and quantal frequency, but evoked excitation is normal because of a homeostatic increase in presynaptic release (Marrus et al., 2004).

For this study, we constructed a *Drosophila melanogaster* stock that exploits partial *GluRIII* loss to study the long-term maintenance of HSP in a single genetic cross. The stock takes advantage of the *GAL4/UAS* expression system and RNA interference (RNAi)-based expression tools (Brand and Perrimon, 1993; Lee and Carthew, 2003; Dietzl et al., 2007; Ni et al., 2008, 2009). Using this stock, we address an open question regarding how Ca_V_2/Cacophony Ca^2+^ channel function gates synaptic homeostasis at the NMJ. Prior studies (ours included), demonstrate that hypomorphic, missense mutations in *cacophony* (*cac*) block homeostatic plasticity (Frank et al., 2006, 2009; Müller and Davis, 2012). Yet here we show that strong knock down of *cacophony* (*cac*) gene function throughout life is not sufficient on its own to impair homeostatic plasticity. We also conduct an RNAi- and electrophysiology-based screen to identify new molecules required for the long-term maintenance of homeostatic plasticity. We document novel roles in synaptic homeostasis for proteins previously implicated in intracellular Ca^2+^ regulation, including Drosophila homologs of Cysteine String Protein (CSP), Phospholipase Cβ (Plc21C), and Gαq.

## Materials and methods

### Drosophila husbandry and stocks

Fruit flies were reared in chambers with temperature control (29°C for RNAi screen; otherwise 25°C). *w^*1118*^* (Hazelrigg et al., 1984) is utilized as a wild-type control unless otherwise indicated. Drosophila stocks carrying various mutant alleles, *GAL4* drivers, or *UAS*-driven transgenes were used. Stocks were either obtained from the Bloomington Drosophila Stock Center (BDSC, Bloomington, IN), the Vienna Drosophila RNAi Center (VDRC, Vienna, Austria), or directly from researchers who generated them. *UAS-RNAi* lines from VDRC (Dietzl et al., 2007) detailed in this manuscript include: *UAS-cac[RNAi]* (VDRC transformant #104168; *cac^*KK101478*^*), *UAS-Csp[RNAi]* (VDRC #34168; *Csp^*GD10571*^*), and *UAS-Plc21C[RNAi]* (VDRC #26558; *Plc21C^*GD11359*^*). *Plc21C* TRiP *UAS-RNAi* lines (Ni et al., 2008, 2009) obtained from BDSC include: *Plc21C^*HMS0600*^*, *Plc21C^*JF01210*^*, *Plc21C^*JF01211*^*. A transgenic wild-type *cac* expression construct is *UAS-cac-eGFP^*786C*^* (Kawasaki et al., 2004). Classical mutant alleles and deficiency stocks include: *GluRIIA^*SP16*^* (Petersen et al., 1997), *Csp^*DG29203*^* (Wishart et al., 2012), *Csp^*EY22488*^* (BDGP gene disruption project, Bellen et al., 2004), *cac^*S*^* (Smith et al., 1998), *cac^*HC129*^* (Kulkarni and Hall, 1987), *Plc21C^*p60A*^* (Weinkove et al., 1999), *Df(2L)BSC4* (J. Deal and K. Cook, BDSC), *Gαq^*1370*^* (Kain et al., 2008), *Gαq^*f04219*^* and *Gαq^221c^* (Banerjee et al., 2006), and *Gαq^28^* (Yao and Carlson, 2010). *GAL4* drivers include *elaV(C155)-GAL4* (Lin and Goodman, 1994), *Scabrous-GAL4* (Budnik et al., 1996), and *BG57-GAL4* (Budnik et al., 1996).

We constructed *UAS-GluRIII[RNAi]* by PCR amplification from genomic DNA, introducing *Xba I* restriction sites, and cloning a tandem duplicated fold-back version of the PCR product into the *pUAST pWiz* vector (Lee and Carthew, 2003). For the PCR amplification step, the *GluRIII* gene PCR primers were as follows: CAF2L: 5′-TCGATCTAGAGATCCTCGAGCGAGGATGGACAGCGGA-3′ and CAF2R: 5′-TTATTCTAGATGATTATCTCGCCAATGATGC-3′. Standard injection procedures were used to generate *UAS-GluRIII[RNAi]* transgenic stocks. A *UAS-GluRIII[RNAi]* insertion on chromosome III was built into a screening stock with the full genotype: *elaV(C155)-GAL4; Scabrous-GAL4/CyO-GFP; BG57-GAL4, UAS-GluRIII[RNAi]/TM6B*. For simplicity, this screening stock is termed *T15* in the manuscript, and balancer chromosomes are not included in its description in the Results Section of the text.

### Electrophysiology and analysis

Wandering third instar larvae were selected for electrophysiological recordings. Sharp electrode recordings were taken from muscle 6 of abdominal segments 2 and 3, as previously described (Davis et al., 1998; Frank et al., 2006). Larvae were dissected in a modified HL3 saline: NaCl (70 mM), KCl (5 mM), MgCl2 (10 mM), NaHCO3 (10 mM), sucrose (115 mM = 3.9%), trehalose (4.2 mM = 0.16%), HEPES (5.0 mM = 0.12%), and CaCl_2_ (0.5 mM, unless otherwise indicated). Data were collected using Axopatch 200B or Axoclamp 900A amplifiers (Molecular Devices, Sunnyvale, CA), digitized using a Digidata 1440A data acquisition system (Molecular Devices), and recorded with pCLAMP 10 acquisition software (Molecular Devices). For presynaptic nerve stimulation, a Master-8 pulse stimulator (A.M.P. Instruments, Jerusalem, Israel) and an ISO-Flex isolation unit (A.M.P. Instruments) were utilized to deliver 1 ms suprathreshold stimuli to the appropriate segmental nerve. The average spontaneous miniature EPSP (mEPSP) amplitude was quantified by measuring the amplitude of ~100–200 individual spontaneous release events per NMJ (unless mEPSP frequency was too low for ~1 min continuous recording, in which case all mEPSPs available were measured). The average per-NMJ mEPSP amplitudes were then averaged for each genotype. The average evoked EPSP amplitude was calculated for each NMJ. Quantal content (QC) was determined for each recording by calculating the ratio of average EPSP and average mEPSP amplitudes. Quantal contents were calculated for each recording and then averaged across NMJs of the indicated genotypes. Where indicated, quantal content was corrected for non-linear summation (Martin, 1955). Selected raw electrophysiological data are included as Supplementary Table 1.

### Immunostaining

Third instar larvae were fileted in HL3 saline. Dissected animals were fixed for 3 min in Bouin's fixative (Ricca Chemical Company, Arlington, TX), washed using standard procedures, and incubated in primary antibodies overnight at 4°C. This was followed by additional washes and a 2-hour incubation in secondary antibody at room temperature. Staining was performed using the following primary antibodies: mouse anti-Synapsin (3C11) 1:50 (Developmental Studies Hybridoma Bank, University of Iowa—DSHB) (Klagges et al., 1996); rabbit anti-Dlg 1:30,000 (Budnik et al., 1996); mouse anti-Brp (nc82) 1:250 (DSHB); rabbit anti-GluRIII 1:4000 (Marrus et al., 2004). The following fluorophore-conjugated antibodies were also used (Jackson ImmunoResearch Laboratories, Inc., West Grove, PA): goat anti-mouse-488 1:1000 (DyLight); goat anti-rabbit-549 1:2000 (DyLight); Alexa-647 goat anti-HRP 1:500. Larval preparations were mounted in Vectashield (Vector Laboratories, Burlingame, CA) and imaged at room temperature using Zen software on a Zeiss 700 LSM mounted on an Axio Observer.Z1 using an EC Plan-Neofluar 40X Oil DIC Objective (aperture 1.30) or an EC Plan-Apochromat 63x Oil DIC Objective (aperture 1.40) (Carl Zeiss Microscopy, Jena, Germany). For each experiment, experimental and control larval preps were stained in the same container, mounted on the same slide, imaged using identical acquisition settings, and analyzed using the same procedure and thresholds.

### Image analyses

Bouton numbers were quantified semi-automatically using the “Spots” function in Imaris x64 v7.6.0 (Bitplane, Zurich Switzerland). Boutons were counted using the anti-Synapsin channel with the XY diameter set at 3 μm. Active Zones were counted using the anti-Brp channel with an XY diameter of 0.3 μm. GluRIII clusters were counted using the anti-GluRIII channel with an XY diameter of 0.5 μm. The threshold was adjusted so that each bouton, active zone, or GluRIII cluster was recognized once. Any errors in automated counting were corrected by hand to arrive at the final value. GluRIII levels and area were assessed using ImageJ 1.48s/Java 1.6.0_24 (64-bit) with Fiji plugins. Z-stack images were compressed using the maximum projection function; an ROI was generated from a mask of the HRP channel and used to define the synapse; a second ROI was hand drawn to exclude any non-synaptic structures in the image; a minimum threshold was set for each channel to eliminate background fluorescence and held consistent within each experiment; the Measure function was used to assess fluorescence intensity and area for each channel [Brp(488), GluRIII(549), HRP(647)]. For each NMJ, total GluRIII fluorescence intensity and synapse coverage was normalized to the synaptic area of the HRP channel and compared between the genotypes analyzed. Cluster area was calculated by dividing the total synaptic area of GluRIII by the number of GluRIII clusters for each synapse imaged.

### RNA extraction and quantitative RT-PCR

For each genotype, 25–30 wandering third instar, female larvae were sorted in HL3 saline, collected on ice and rinsed in HL3 with excess saline pipetted off before homogenization. Larvae were homogenized in 200 μL TRIzol reagent (Ambion, Life Technologies) using disposable plastic micropestles. Total homogenate was then brought to 1 mL by addition of 800 μL TRIzol, tubes were vigorously inverted, and the resultant homogenate was frozen at −80°C until all genotypes were prepared. Samples were allowed to thaw on ice and incubated for 5 min at room temperature before proceeding with RNA isolation using the manufacturer's instructions with the following changes: Following isopropanol addition, samples were placed at −20°C for precipitation, and two ethanol wash cycles were used. Samples were quantified using a Nanodrop (Thermo Scientific). Reverse transcription was done using iScript (Bio-Rad) according to manufacturer's instructions using 1 μg of total input. Quantitative PCR (Bio-Rad CFX96) was done using SYBR Green reagent (Bio-Rad) with a 10 μL final reaction volume and technical replicates in triplicate. Final primer concentrations were 250 nM. For *cac*, exon spanning primers were designed with the following sequences: cac-F 5′-cgggaacgagagttgtacg-3′ and cac-R 5′-actggagatggcagtacacg-3′ (this study), and RpL32-F 5′-atgaccatccgcccagcatac-3′ and RpL32-R 5′-ctgcatgagcaggacctccag-3′ (PK Geyer Lab, University of Iowa). Primer piloting was done using stepwise dilutions of iScript product to verify PCR efficiency. Final quantification was done using iScript product diluted to 1:444.4 in the final PCR. Melt curve analysis verified presence of a single product for all reactions.

### Statistical analyses

Statistical significance was assessed either by Student's *T*-Test, comparing an experimental data set and a control data set, or One-Way ANOVA with Tukey's *post-hoc* test across multiple data sets, as appropriate. Specific *p* value ranges are given in the text, figures, and figure legends, with *p* < 0.05 marked as significant (^#^*p* = 0.05; ^*^p < 0.05; ^**^p < 0.01; ^***^p < 0.001). For some *p*-values that trend toward statistical significance (0.05 < *p* < 0.1), specific values are given as indicated. The values reported or plotted on bar graphs are mean ± SEM.

## Results

### Homeostatic challenge: *GluRIII* gene knock down by RNAi

**Figure 1:**
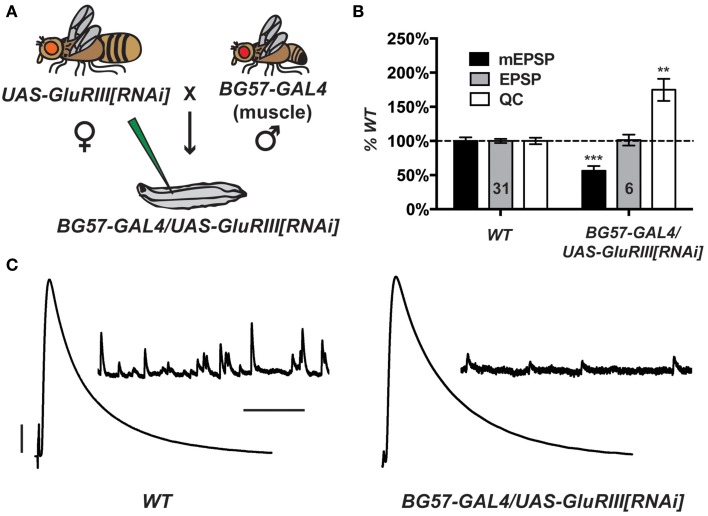
**Postsynaptic *GluRIII* gene knock down induces robust homeostatic compensation. (A)** Crossing scheme. NMJs from F1 larvae (genotype *BG57-GAL4/UAS-GluRIII[RNAi]*) are subjected to electrophysiological analyses. **(B)** Quantal size (miniature excitatory postsynaptic potentials, mEPSP) is decreased for *BG57-GAL4/UAS-GluRIII[RNAi]* larvae (***p < 0.001, Student's *T*-Test). Evoked potentials (excitatory postsynaptic potentials, EPSP) are normal because of a homeostatic increase in quantal content (QC) (**p < 0.01). **(C)** Representative electrophysiological traces. Scale bars for EPSPs (mEPSPs): 5 mV (1 mV); 50 ms (1000 ms).

Partial knock down of *GluRIII* gene function should induce a significant decrease in NMJ quantal size and a homeostatic increase in NMJ quantal content (Marrus et al., 2004). Using the Drosophila *pWiz* vector (Lee and Carthew, 2003), we created a transgene to target the *GluRIII* gene for functional knock down by RNAi (see Materials and Methods). From a *pWiz-UAS-GluRIII[RNAi]* clone, we generated *UAS-GluRIII[RNAi]* transgenic Drosophila stocks and crossed them to stocks harboring the muscle-specific *GAL4* driver, *BG57-GAL4* (Budnik et al., 1996) (Figure 1A). We then analyzed the larval cross progeny by electrophysiology to assess the efficiency of glutamate receptor subunit knock down.

Postsynaptically driven *BG57-GAL4* >> *UAS-GluRIII[RNAi]* yields a robust homeostatic challenge to NMJ function (Figures 1B,C) (See Supplementary Table 1 for selected raw electrophysiological data throughout the manuscript). NMJ effects are similar to the published *GluRIII* hypomorphic condition (Marrus et al., 2004). Compared to controls, *BG57-GAL4/UAS-GluRIII[RNAi]* NMJs show drastically decreased quantal amplitude (mEPSP = 0.81 ± 0.04 mV for wild-type control vs. 0.46 ± 0.05 mV for *GluRIII* knock down, *p* < 0.001, *T*-Test) and frequency (4.2 ± 0.2 Hz for control vs. 0.8 ± 0.1 Hz for *GluRIII* knock down, *p* < 0.001, *T*-Test). Despite these decreases in spontaneous miniature neurotransmission, evoked neurotransmission is normal (EPSP = 33.2 ± 1.0 mV for control vs. 33.6 ± 2.7 mV for *GluRIII* knock down) because of a homeostatic enhancement of quantal content (*QC* = 43.8 ± 2.1 for control vs. 76.6 ± 7.1 for *GluRIII* knock down, *p* < 0.01, *T*-Test) (Figures 1B,C).

By meiotic recombination, we placed a *UAS-GluRIII[RNAi]* transgene on chromosome III in *cis* with the *BG57-GAL4* driver. To increase the potential of this stock as a genetic tool to study NMJ homeostasis, we augmented it with two presynaptic *GAL4* drivers. We chose neuronal drivers *elaV(C155)-GAL4* (Lin and Goodman, 1994) and *Scabrous-GAL4* (Budnik et al., 1996). Multiple presynaptic drivers were used to enhance the efficiency of RNAi in neurons. For simplicity, we refer to the new stock incorporating all drivers as *T15* (trans-synaptic). The genotype of *T15* is *elaV(C155)-GAL4*; *Sca-GAL4*; *BG57-GAL4, UAS-GluRIII[RNAi]* (chromosomes X; II; III, balancer chromosomes omitted from this genotype). We also generated a control stock, containing the same *GAL4* drivers, but not the *UAS-GluRIII[RNAi]* transgene.

**Figure 2:**
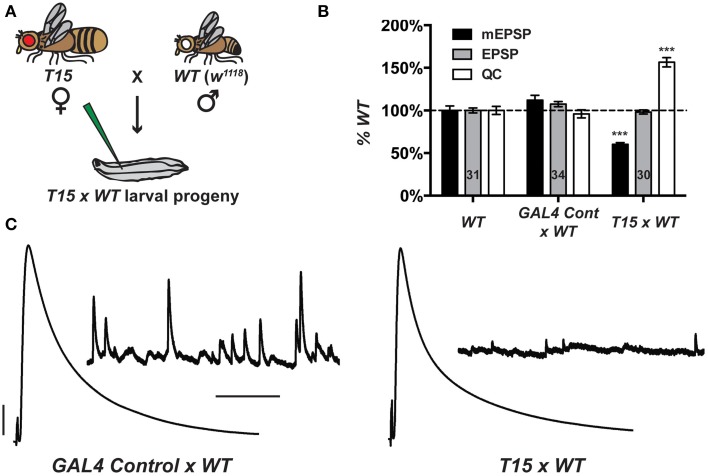
**The *T15* stock induces robust homeostatic compensation. (A)** *T15 × WT* crossing scheme. The genotype for *T15* is (chromosomes X; II; III): *elaV(C155)-GAL4; Scabrous-GAL4; BG57-GAL4, UAS-GluRIII[RNAi]*. **(B)** *T15 × WT* larval NMJs have decreased quantal size (***p < 0.001, *T*-Test vs. *WT*). Evoked potentials are normal because of a homeostatic increase in QC (***p < 0.001). A control stock with only GAL4 drivers behaves similarly to *WT*. **(C)** Representative electrophysiological traces. Scale bars for EPSPs (mEPSPs): 5 mV (1 mV); 50 ms (1000 ms).

When *T15* females are crossed to wild-type males (herein *T15* × *WT*, Figure 2A), the larval progeny exhibit starkly diminished NMJ quantal size and frequency, and a homeostatic increase in NMJ quantal content (Figures 2B,C). By contrast, when *GAL4* driver control females are crossed to wild-type males (herein *GAL4 Cont* × *WT*), the NMJ electrophysiological profile of larval progeny is largely similar to that of wild-type NMJs (Figures 2B,C). The *GAL4* drivers or genetic background may induce a slight increase in quantal size (Figure 2B). However, this increase is not statistically significant, and the data show that the presence of presynaptic *GAL4* drivers exerts no adverse effects on evoked NMJ excitation (Figures 2B,C).

### *GluRIII* knock down does not grossly alter synapse development

**Figure 3:**
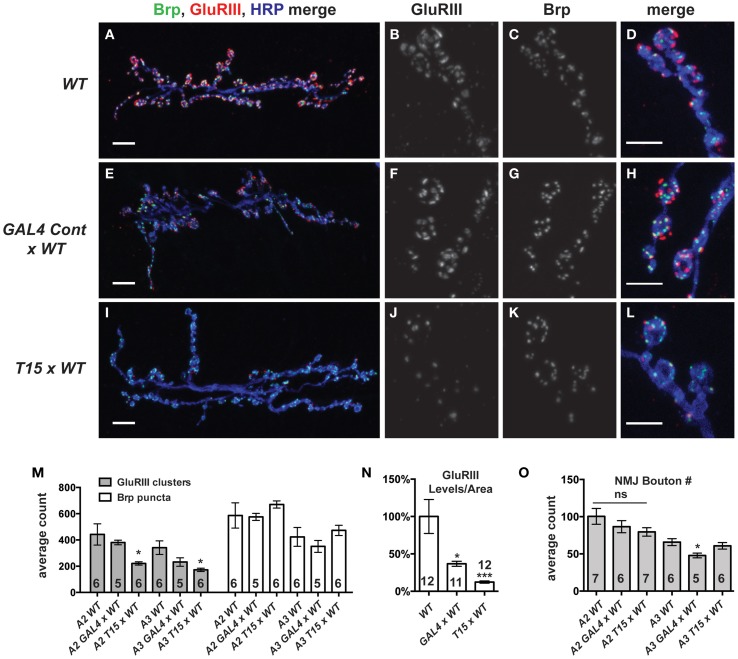
**GluRIII glutamate receptor subunits are dramatically decreased in the T15 line. (A-L)** Immunostaining of wild type (*WT*), *GAL4 Cont × WT*, and *T15 × WT* NMJs with antibodies against GluRIII (red), Bruchpilot (Brp, green), and HRP (blue). A, E, and I show 40X images (scale bars, 10 μm) of muscle 6/7 NMJs from wandering third instar larvae. **(B–D,F–H,J–L)** Panels show various channels of 60X images (scale bars, 5 μm) of NMJs. **(M)** Quantification of the number of presynaptic active zones (marked by Brp) and GluRIII clusters at the muscle 6/7 synapse of segments A2 and A3 (*p < 0.05). **(N)** Calculation of total GluRIII levels per unit of synapse area. This measure takes into account both GluRIII cluster size and GluRIII intensity (see text for individual values; see Materials and Methods for details; *p < 0.05 compared to WT; ***p < 0.001). **(O)** Quantification of the number of boutons at segment A2 and A3 muscle 6/7 NMJs. *n* ≥ 6 NMJs stained for each condition.

We wished to examine the effects of these NMJ drivers and the *UAS-GluRIII[RNAi]* transgene on synapse development. We imaged larval NMJs by immunofluorescence microscopy. We utilized an anti-GluRIII antibody to examine glutamate receptors (Marrus et al., 2004). As expected, *T15* × *WT* NMJs show a marked decrease in anti-GluRIII NMJ staining compared to wild-type controls or *GAL4* × *WT* controls (Figures 3A–N). We note several aspects of anti-GluRIII staining that are diminished at *T15 × WT* NMJs. First, there is a 50% decrease in the number of anti-GluRIII puncta compared to wild-type control NMJs for muscles 6 and 7 (Figure 3M, *p* < 0.05, *T*-Test for both segments A2 and A3). In addition to this reduction in cluster number, we observed that individual GluRIII cluster size was greatly reduced (41.6 ± 3.5% compared to wild-type controls, *p* < 0.001, *T*-Test). Further analysis of digital immunofluorescence images reveals that the average intensity of each anti-GluRIII pixel is also decreased (82.3 ± 3.9% for the *T15 × WT* condition compared to wild-type controls, *p* = 0.03, *T*-Test). This intensity decrease is small. However, when considered in combination with the reductions in cluster number and size, we estimate an 88% decrease in GluRIII NMJ protein per unit of synapse area in *T15 × WT* larvae compared to wild-type larvae (Figure 3N, *p* < 0.001, *T*-Test; total fluorescence intensity, normalized for synapse area, see Materials and Methods). As expected with RNAi-mediated knock down, this is not a complete loss of GluRIII protein. Importantly these changes are consistent with our electrophysiological data for *T15 × WT* showing significantly decreased quantal size (reduced intensity/pixel and cluster size) and frequency (reduced cluster number).

Surprisingly, the presence of the *GAL4* drivers (or the genetic background of the driver line stock) also appears to diminish GluRIII protein level (per unit of synapse area) in *GAL4 × WT* compared to wild-type controls (Figure 3N, *p* < 0.05). We are not certain why GluRIII levels are down in *GAL4 × WT*. Nevertheless, by electrophysiology, this depression is clearly not severe enough to diminish quantal size or evoked excitation (Figure 2B)—though it could explain a partial decrease in quantal frequency compared to *WT* (Supplementary Table 1). Nevertheless, the amount of GluRIII protein per unit synapse area in *GAL4 × WT* is about three times greater than the amount measured in *T15 × WT* (Figure 3N, *p* < 0.001).

We wished to examine other basic elements of synapse structure. To do this, we stained NMJs with an anti-Bruchpilot (Brp) antibody to count the number of presynaptic active zones (Wagh et al., 2006) or with anti-Discs Large (Dlg) (Budnik et al., 1996) and anti-Synapsin (Syn) (Klagges et al., 1996) antibodies to count the number of synaptic boutons. For synapses on both segments A2 and A3, neither active zone counts (Figure 3M, Brp puncta) nor bouton counts (Figure 3O) are significantly different when comparing *T15 × WT* NMJs vs. wild-type NMJs. These findings are interesting, considering the fact that *T15 × WT* NMJs have fewer GluRIII clusters apposed to the presynaptic active zone (Figure 3M). Collectively, our data show that *T15 × WT* NMJs have starkly decreased GluRIII levels, but otherwise grossly normal synapse growth and morphology.

### RNAi- and electrophysiology-based screening

**Figure 4:**
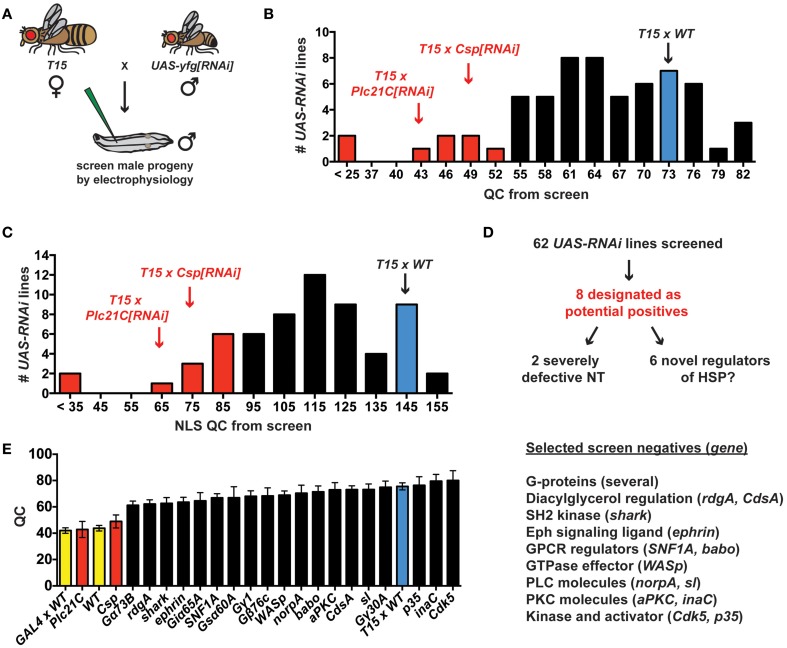
**An RNAi- and electrophysiology-based screen for homeostatic factors. (A)** Crossing scheme for screen *T15 × UAS-yfg[RNAi]* (“your favorite gene”). For *UAS-RNAi* lines on chromosomes II or III, male progeny are examined by electrophysiology because dosage compensated *elaV(C155)-GAL4/Y* male progeny should have a higher dose of presynaptic GAL4 than *elaV(C155)-GAL4*/+ female siblings. **(B)** Distribution of QC values from screened larvae. Eight *T15 × UAS-RNAi* crosses yield a QC smaller than two standard deviations below *T15 × WT* (red bars). **(C)** When QC is corrected for non-linear summation (NLS), twelve *T15 × UAS-RNAi* line crosses yield an NLS corrected QC (NLS QC) smaller than two standard deviations below WT (red bars). **(D)** Schematic to sort potential positives for follow-up analyses. **(E)** Negative data for some genes in the screen. Where a specific gene name is listed, the data represent the QC for *T15 × UAS-yfg[RNAi]*. Underlying data for displayed screen negatives has average evoked potentials >30 mV (not shown, but *WT* control EPSP = 33.2 ± 1.0 mV) and *QC* > 60.

The prior electrophysiological data illustrate the potential utility of *T15* as a genetic tool to study HSP. If *T15* animals are crossed to animals bearing an effective *UAS-RNAi* transgene, the target gene should be knocked down in larval progeny both presynaptically (due to *elaV(C155)-GAL4* and *Sca-GAL4* driving *UAS-RNAi* of the target gene in neurons) and postsynaptically (due to *BG57-GAL4* driving *UAS-RNAi* of the target gene in muscle)—all in the context of a homeostatic challenge to NMJ function due to *GluRIII* gene knock down. To employ *T15* for this purpose, we executed a small-scale RNAi- and electrophysiology-based screen. We chose 62 *UAS-RNAi* stocks on chromosomes II and III (Dietzl et al., 2007; Ni et al., 2008, 2009). The screen was biased: we targeted genes encoding factors potentially required for proper presynaptic Ca^2+^ entry and handling, G-protein signals (which could impinge upon calcium channel function), factors known to regulate general synaptic functions, and possible trans-synaptic signaling molecules. To conduct the screen, we crossed *T15* females with *UAS-RNAi* males and recorded from the NMJs of male larval progeny (Figure 4A). Male progeny were chosen because they should have a stronger dose of X-linked *elaV(C155)-GAL4* than female progeny.

*T15* × *WT* NMJs have significantly elevated quantal content compared to non-transgenic wild-type controls (Figures 2B, 4B). In analyzing the *T15 × UAS-RNAi* screen data, we considered quantal content as a measure of presynaptic release. We identified eight *T15* × *UAS-RNAi* crosses that yielded progeny with a low NMJ quantal content (*QC* < 52, Figure 4B). For all eight, the level of presynaptic release is indistinguishable (or lower) than that of unchallenged, wild-type NMJs, potentially indicative of a block in homeostatic compensation. Considering the distribution of QC for all of the *UAS-RNAi* crosses from the screen, this level of presynaptic release is also about two standard deviations smaller than *T15 × WT* controls. We wished to ensure that our synaptic release analysis was accurate and restrictive. Therefore, we also applied a correction to our calculations of quantal content to account for effects of non-linear summation (NLS) when recording synaptic voltages (Martin, 1955). Considering the distribution of NLS corrected QC for all of the *UAS-RNAi* crosses from the screen, an NLS corrected *QC* < 85 is two standard deviations smaller than *T15* × *WT* controls. Twelve *T15 × UAS-RNAi* crosses fall below this NLS QC threshold, including all eight identified by the prior cutoff (Figure 4C). We chose to focus initial follow-up efforts on the eight potential positives that showed a low quantal content by both criteria (Figure 4D).

We identified eight potential hits out of 62 *UAS-RNAi* stocks, which is a very high positive rate for a screen (12.9%). However, the screen is biased, and it is not surprising that several pre-selected factors could impair synapse function. Nevertheless, most *T15 × UAS-RNAi* crosses do not yield progeny with diminished NMJ QC. Several screen negatives with EPSP > 30 mV and *QC* > 60 are shown (Figure 4E). It is possible the corresponding target genes are not involved in synaptic homeostasis. However, it is also possible that conditions in this experiment do not yield sufficient gene knock down to reveal a homeostatic phenotype. One interesting example to consider in this regard is Drosophila *ephrin*. Prior work demonstrates that a presynaptic signaling system consisting of the Eph receptor tyrosine kinase and the cytoplasmic guanine exchange factor Ephexin is needed for the long-term maintenance of HSP (Frank et al., 2009). Drosophila Ephrin is a top candidate ligand for Drosophila Eph in this process, but the RNAi data from our screen do not support this conclusion (Figure 4E).

A low QC value from a screened *T15* × *UAS-RNAi* line could reflect a genuine defect in synaptic homeostasis. It could also simply reflect a decrease in neurotransmission when the targeted gene is knocked down in NMJ tissues. We note that two of the eight *UAS-RNAi* lines identified appear to cause severe neurotransmission defects (Figures 4B–D), suggesting synapse dysfunction independent of possible defects in homeostatic plasticity (*QC* < 25 when crossed to *T15*; NLS *QC* < 35 when crossed to *T15*). One of these two RNAi lines targets Drosophila *cacophony* (*cac*)—a positive control RNAi line included in the screen that targets the α1a pore-forming subunit of Ca_V_2 channels. Given previous studies examining Ca_V_2 in neurotransmission and NMJ homeostasis (Frank et al., 2006, 2009; Tsurudome et al., 2010; Müller and Davis, 2012), this result is not surprising. However, using *T15*, we are able to garner new information about Cac/Ca_V_2 and its role in synaptic homeostasis (see below). Finally, we also report initial characterizations of two novel homeostatic genes, Drosophila *Csp* and *Plc21C* (Figures 4B,C). Our screening and characterizations of other genes are ongoing, and we will report further characterizations elsewhere.

### Cacophony knock down by RNAi impairs neurotransmission but not homeostatic plasticity

Ca_V_2-type voltage-gated Ca^2+^ channels are critical for presynaptic neurotransmission. For many vertebrate and invertebrate synapses, Ca_V_2 channels also gate homeostatic modulations of neurotransmission (Frank, 2014b). In Drosophila, homozygous, partial loss-of-function missense mutations like *cac^S^* block the homeostatic potentiation of transmitter release at the NMJ after glutamate receptor impairment (Frank et al., 2006, 2009; Müller and Davis, 2012). By contrast, *cac* null mutants arrest as embryos (Kulkarni and Hall, 1987; Kurshan et al., 2009). Therefore, it is unknown if *cac* mutant impairment of HSP is due to partial loss of channel function—or if amino-acid substitutions such as the one encoded by *cac^S^* (F1029I, transmembrane domain III S6) (Smith et al., 1998) impair specific functions or interactions critical to homeostatic signaling. Intriguingly, *cac^S^/+* heterozygotes display partial defects in courtship song behavior (Smith et al., 1998) and the execution of synaptic homeostasis (Frank et al., 2006, 2009). These data are consistent with the possibility that missense *cac* mutations may exert antimorphic effects on synaptic function.

Knocking down *cac* gene activity by RNAi allows one to test whether a simple reduction of Ca_V_2 function suffices to impair synaptic homeostasis. Efficient RNAi-mediated *cac* knock down also circumvents possible antimorphic effects associated with genetic mutations (but not potential effects due to haploinsufficiency). For the screen and subsequent analyses here, we used a *UAS-cac[RNAi]* transgene to knock down *cac* gene function (*cac^KK101478^*, see Materials and Methods) (Dietzl et al., 2007). By primary target sequence, *cac^KK101478^* is not predicted to induce off-target gene knock down. By quantitative RT-PCR (qPCR), we find that driving this transgene under simultaneous control of the neuronal drivers *elaV(C155)-GAL4 and Sca-GAL4* diminishes total levels of *cac* mRNA by ~50% vs. a control utilizing just the drivers (See Materials and Methods).

We prepared mRNA for qPCR using whole larvae. Therefore, Cac protein loss in motor neurons and at synapses could be even greater than qPCR measurements. We tested the effectiveness of *UAS-cac[RNAi]* on *cac* gene function in two different ways. First, we crossed transgenically rescued *cac* null females *elaV(C155)-GAL4, cac^HC129^*;; *UAS-cac-eGFP^786c^* × *UAS-cac[RNAi]* males (or *WT* males for control). This *UAS-cac[RNAi]* cross (but not the *WT* control cross) kills all progeny, with only a small number of adults reaching the pupal eclosion stage and then either arresting during the process of eclosion or getting stuck in the food shortly after eclosing (not shown). Second, we find that presynaptic expression of *cac^KK101478^* starkly diminishes evoked neurotransmission (*elaV(C155)-GAL4*, *Sca-GAL4* >> *UAS-cac[RNAi]*: EPSP = 8.5 ± 1.1 mV; *QC* = 8.4 ± 1.5, *n* = 8) compared to a wild-type control (WT: EPSP = 33.2 ± 1.0 mV, *QC* = 43.8 ± 2.1, *n* = 31). This diminishment of evoked neurotransmission is similar in severity to a *cac^S^* mutant (Frank et al., 2006), and it suggests a significant loss of Cac protein at the NMJ. By contrast, muscle expression of *cac^KK101478^* (*BG57-GAL4 >> UAS-cac[RNAi]*) has no adverse effect on evoked neurotransmission (EPSP = 37.5 ± 2.0 mV; *QC* = 53.2 ± 2.9, *n* = 8).

**Figure 5:**
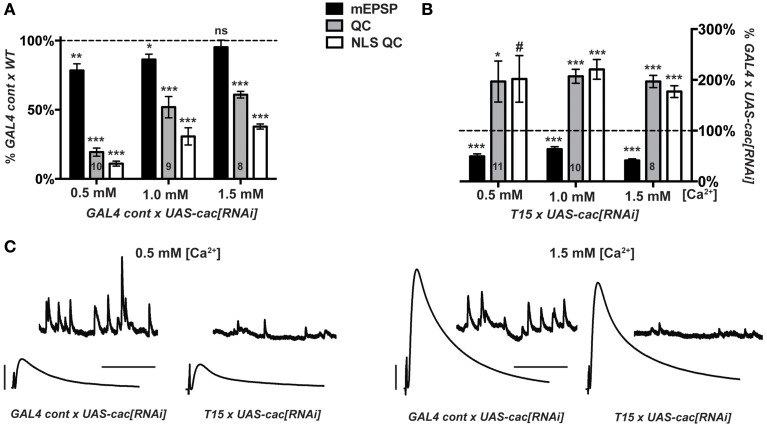
**Knock down of *cacophony* gene function does not impair synaptic homeostasis. (A,B)** mEPSP (black), QC (gray), NLS QC (white). (^#^*p* = 0.05; *p < 0.05; **p < 0.01; ***p < 0.001, *T*-test compared to control at 100% dotted line) **(A)** Diminished baseline neurotransmission for *GAL4 control × UAS-cac[RNAi]* larvae is consistent with *cac* gene knock down. Diminished release occurs across a range of extracellular [Ca^2+^]. **(B)** *T15 × UAS-cac[RNAi]* larvae show robust homeostatic compensation compared to *GAL4 control × UAS-cac[RNAi]* larvae. As expected, the *T15* line induces a marked diminishment of quantal size (mEPSP). The NMJ responds with a robust increase in release. This response is observed across a range of extracellular calcium concentrations, and the same result holds whether or not QC is corrected for non-linear summation. **(C)** Representative electrophysiological traces. Scale bars for EPSPs (mEPSPs): 5 mV (1 mV); 50 ms (1000 ms).

We crossed either the *T15* stock or the aforementioned pre- and post-synaptic *GAL4* driver control stock (Figure 2) to *UAS-cac[RNAi]*. For *GAL4 Cont × UAS-cac[RNAi]* progeny, NMJ evoked excitation and quantal content are markedly decreased compared to *GAL4 Cont × WT* controls (Figure 5A). This is true across a range of extracellular Ca^2+^ concentrations, though perhaps a bit less pronounced at physiological [Ca^2+^] (1.5 mM) (Figure 5A). As expected, for *T15* × *UAS-cac[RNAi]* larval progeny, there is a significant decrease in NMJ quantal size (mEPSP) compared to *GAL4 Cont × UAS-cac[RNAi]* larval progeny (Figure 5B). However, *T15* × *UAS-cac[RNAi]* NMJ EPSP amplitude is no different than control cross *UAS-cac[RNAi]* progeny (Figure 5C). This is due to a homeostatic enhancement in presynaptic quantal content (Figure 5B). This result holds for extracellular Ca^2+^ concentrations that permit a range of presynaptic release: 0.5, 1.0, and 1.5 mM. For each condition, homeostatic compensation of presynaptic release remains robust (Figure 5B).

Our data suggest diminishment of *cac* gene function itself is not sufficient to block homeostatic potentiation of function. Nor is extracellular calcium concentration a factor. If Cac/Ca_V_2 levels are simply diminished—but the copies of Cac α1a subunits present are wild-type copies—homeostatic plasticity is intact. By contrast, the presence of function-impairing Ca_V_2 α1a subunits due to amino-acid substitution appears sufficient to block the induction and maintenance of homeostatic plasticity (Frank et al., 2006, 2009; Müller and Davis, 2012).

### Cysteine string protein mutations block the long-term maintenance of synaptic homeostasis

CSP is a conserved synaptic protein in the DnaJ family of chaperones. For both vertebrates and invertebrates, CSP executes functions that promote viability, coordinated locomotion, neuroprotection, and evoked neurotransmitter release (Zinsmaier, 2010). At the Drosophila NMJ, synaptic functions of CSP appear related to Ca^2+^ regulation. In *Csp* loss-of-function mutants, resting intra-terminal Ca^2+^ levels are altered (Dawson-Scully et al., 2000; Bronk et al., 2001; Dawson-Scully et al., 2007), and neurotransmission defects can be suppressed by high [Ca^2+^]_e_ or repetitive nerve stimulation (Dawson-Scully et al., 2000; Bronk et al., 2001). The fact that a *UAS-Csp[RNAi]* line emerged from our screen was somewhat surprising because a prior genetic screen demonstrated that a *Csp* mutation does not impair homeostatic plasticity when the NMJ is challenged with PhTox application (Goold and Davis, 2007; Dickman and Davis, 2009). However, this previous screen specifically examined factors for a role in the short-term induction of homeostatic plasticity, not its long-term maintenance.

**Figure 6:**
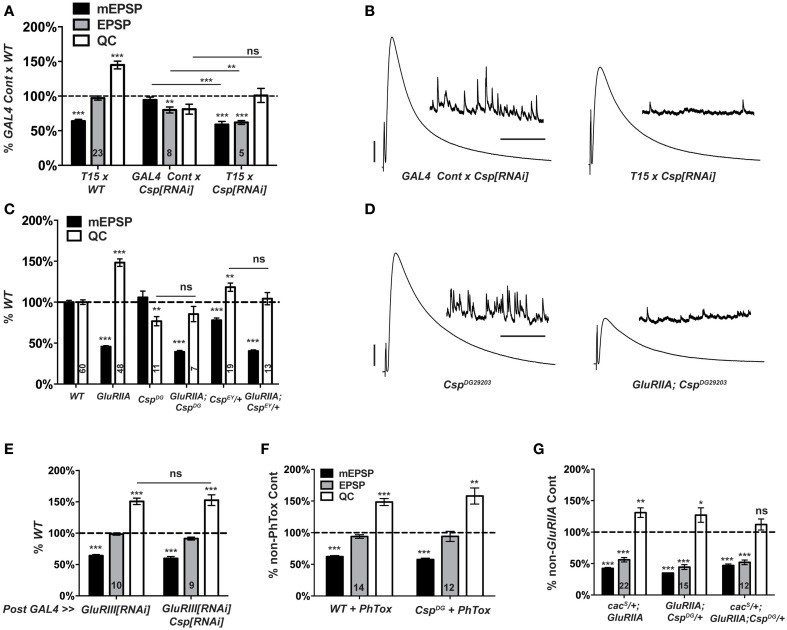
**C*sp* is required for long-term homeostatic compensation. (A)** *T15 × UAS-Csp[RNAi]* shows a failure to upregulate quantal content compared to its *GAL4*-driven *UAS-Csp[RNAi]* control (ns, *p* = 0.15). Knock down of *Csp* shows a slight impairment in evoked neurotransmission (EPSP) compared to control (**p < 0.01). **(B)** Representative electrophysiological traces show the failure of *T15 × UAS-Csp[RNAi]* larvae to maintain evoked potentials at control levels. **(C)** Homozygosity for the C*sp^DG29203^* allele or heterozygosity for the *Csp^EY22488^* allele block homeostatic upregulation of quantal content compared to their respective non-*GluRIIA^SP16^*genetic controls (ns, *p* = 0.44 and *p* = 0.14, respectively). **(D)** Representative traces show a failure of homeostatic compensation for *GluRIIA^SP16^*, *Csp^DG29203^*. **(E)** Postsynaptic knock down of *Csp* function leaves homeostatic plasticity intact. **(F)** Acute homeostatic compensation is intact in *Csp^DG29203^* as evidenced by the elevated quantal content in response to philanthotoxin-433 (PhTox) application (**p < 0.01). **(G)** A doubly heterozygous combination of *Csp^DG29203^*/+ and *cac^S^/+* shows a homeostatic block in the *GluRIIA^SP16^* background because of a failure to increase quantal content over *cac^S^*/+; *Csp^DG29203^*/+ controls (ns, *p* = 0.24). By contrast, the single heterozygous mutations retain partial homeostatic compensatory capacity. Scale bars for EPSPs (mEPSPs): 5 mV (1 mV); 50 ms (2000 ms). (ns, *p* > 0.05; *p < 0.05; **p < 0.01; ***p < 0.001).

Our screen found no elevation of NMJ quantal content when comparing *T15 × UAS-Csp[RNAi]* larval progeny vs. wild-type larvae (Figure 4). This result indicates a possible role for CSP in homeostatic plasticity, although it could also be consistent with expected *Csp* defects in neurotransmission. In a follow-up test, we found that *T15 × UAS-Csp[RNAi]* NMJ quantal content is not significantly different than *GAL4* driver control × *UAS-Csp[RNAi]* NMJ quantal content (Figure 6A). This result suggests a bona fide defect in synaptic homeostasis (Figures 6A,B).

To follow up on the RNAi screen with genetic mutations, we examined two transposon insertion-induced *Csp* alleles, *Csp^DG29203^* and *Csp^*EY22488*^*. *Csp^*DG29203*^* disrupts the 5′ UTR of *Csp*, and genetic evidence suggests that it is hypomorphic (Wishart et al., 2012). *Csp^*EY22488*^* is inserted in an intronic region of *Csp*. It causes significant homozygous lethality and appears to be a strong mutation genetically (data not shown). We generated two double mutant combinations: *GluRIIA^*SP16*^*; *Csp^*DG29203*^*, and *GluRIIA*; *Csp^*EY22488*^/+*. The *GluRIIA^*SP16*^* deletion mutation induces a marked decrease in quantal size and a homeostatic increase in quantal content compared to wild-type controls (Figure 6C) (Petersen et al., 1997). By contrast, for the double mutant combinations, synaptic homeostasis is impaired: there is no significant increase in release compared to *Csp* mutants alone (Figure 6C). As a result, evoked postsynaptic excitation is stunted (Figure 6D). Interestingly, the *Csp^*EY22488*^/+* and *Csp^*DG29203*^* genetic conditions display somewhat different baseline electrophysiology, in terms of both quantal size and quantal content (Figure 6C). These differences could be due to different genetic backgrounds or *Csp* allelic strength. Nevertheless, both alleles block homeostatic compensation in response to *GluRIIA* gene loss (Figures 6C,D).

The vast majority of research published about Drosophila CSP demonstrates that it localizes to presynaptic terminals. However, it has also been reported that Drosophila muscles can express low levels of CSP (Eberle et al., 1998). To test whether CSP's function in synaptic homeostasis could reside in the postsynaptic compartment, we crossed the muscle driver stock *BG57-GAL4, UAS-GluRIII[RNAi]* to *UAS-Csp[RNAi]*. Knocking down *Csp* gene function in the muscle leaves synaptic homeostasis intact (Figure 6E); this result is consistent with a presynaptic role for CSP in synaptic homeostasis.

Collectively, our data demonstrate that *Csp* function is needed for the long-term maintenance of homeostatic plasticity. To double check whether *Csp* could also be required for the short-term induction of homeostatic plasticity, we applied PhTox to *Csp^*DG29203*^* mutant NMJs. By this assay, the rapid induction of homeostatic plasticity remains intact (Figure 6F), consistent with the previously reported results (Goold and Davis, 2007; Dickman and Davis, 2009). Therefore, CSP appears to be needed for the sustained expression of homeostatic plasticity throughout development, not its short-term induction.

### A *Csp* and *cac* mutant combination blocks HSP

*cac^S^/+*; *GluRIIA* double mutant NMJs have partially impaired synaptic homeostasis (Frank et al., 2006) (and Figure 6G). As discussed above, this partial impairment could be due to an antimorphic allelic effect. The *cac^S^/+* genetic condition has been previously exploited to characterize second-site factors that could potentially execute homeostatic plasticity in conjunction with Ca_V_2 by searching for genetic interactions with *cac^S^* (Frank et al., 2009; Wang et al., 2014). In the sensitized *cac^S^/+* genetic background, we find that *cac^S^/+*; *GluRIIA*; *Csp^*DG29203*^/+* NMJs have completely blocked synaptic homeostasis—i.e., there is no increase in quantal content compared to the *cac^S^/+*;; *Csp^*DG29203*^/+* genetic control condition (Figure 6G). A strong double heterozygous phenotype could be consistent with Cac and CSP both functioning within synaptic homeostasis, either in a linear signaling pathway or in parallel processes. However, it could also reflect a summation of separate, partial defects in HSP for both *cac^S^*/+; *GluRIIA* NMJs and *GluRIIA; Csp^*DG29203*^*/+ NMJs (Figure 6G).

### Loss of PLCβ function impairs synaptic homeostasis

In designing our screen, we postulated that lipid signaling at the synapse could affect NMJ homeostasis. Canonically, Gαq-GTP (together with Gβγ G-proteins) activates PLCβ. In turn, PLCβ cleaves phosphatidylinositol 4,5-bisphosphase (PIP_2_) into diacylglycerol (DAG) and inositol triphosphate (IP_3_) (Tedford and Zamponi, 2006; Philip et al., 2010; Kadamur and Ross, 2013). These molecules are known to influence neurotransmission in several ways at many synapses, including the NMJ (Goni and Alonso, 1999; Cremona and De Camilli, 2001; Peters et al., 2001; Wu et al., 2002; Rohrbough and Broadie, 2005; Huang et al., 2006). To examine this pathway in the context of NMJ homeostasis, we considered a Drosophila PLCβ homolog gene, *Plc21C*—among other genes, some of which showed no phenotype (Figure 4E).

**Figure 7:**
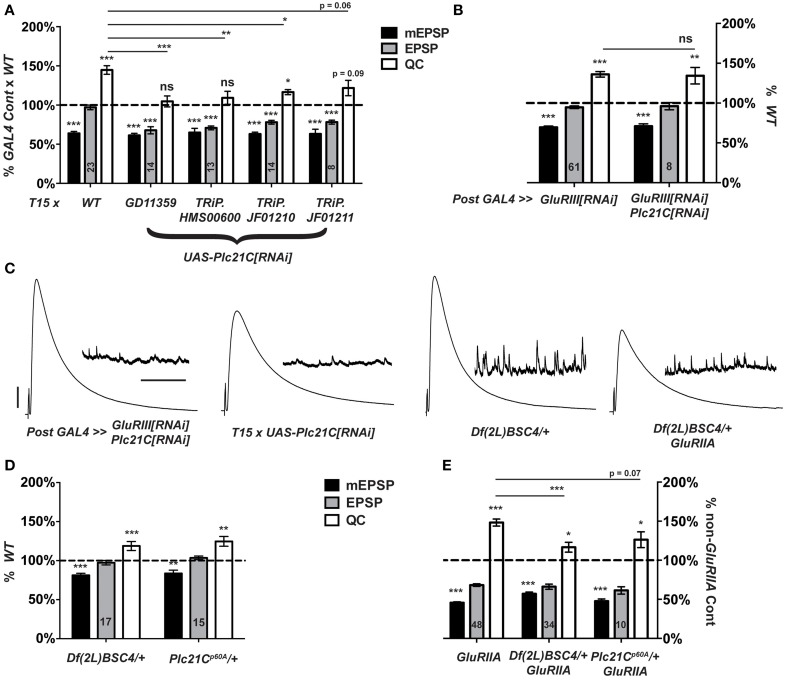
**Presynaptic *Plc21C* is required for homeostatic compensation. (A)** *T15*-mediated RNAi knock down of *Plc21C* results in a failure to maintain the EPSP at control levels (***p < 0.001). Concerning homeostatic compensation, *T15 × TRiP.JF01210* is the only cross to show increased quantal content compared to its *GAL4* cross control (*p = 0.04), but even this quantal content was still significantly depressed compared to the *T15 × WT* control (*p < 0.05, One-Way ANOVA including all crosses in dataset, with Tukey's *post-hoc*). **(B)** Postsynaptic knock down of *Plc21C* does not affect homeostatic compensation: the EPSP is maintained (ns, *p* = 0.45) at control levels because the quantal content is upregulated (**p < 0.01). **(C)** Representative traces show normal neurotransmission upon postsynaptic knock down of *GluRIII* and *Plc21C* or a heterozygous loss of *Plc21C* via use of the deficiency chromosome *Df(2L)BSC4*. However, evoked release is not maintained upon *T15*-mediated knock down of *Plc21C* or the heterozygous loss of *Plc21C* in a *GluRIIA^*SP16*^* mutant background. **(D)** Evoked amplitudes (EPSPs) are unaltered upon heterozygous loss of *Plc21C* by use of the deficiency *Df(2L)BSC4* or *Plc21C^*p60A*^* allele though both do show a small, yet significant decrease in the amplitude of mEPSP events (***p < 0.001 and **p < 0.01, respectively). **(E)** Quantal content is minimally increased in the *GluRIIA^*SP16*^* background upon heterozygous loss of *Plc21C* compared to their respective genetic controls (*p < 0.05). For the *Df(2L)BSC4* deficiency, the increased quantal content does not reach the level of increase found in *GluRIIA^*SP16*^* (***p < 0.001, One-Way ANOVA, Tukey's *post-hoc*). This is indicative of a partial impairment in homeostatic compensation. Scale bars for EPSPs (mEPSPs): 5 mV (1 mV); 50 ms (2000 ms).

When *T15* is crossed to a *UAS-Plc21C[RNAi]* transgenic line (Dietzl et al., 2007), larval progeny have significantly lower NMJ quantal content than *T15 × WT* controls (Figures 4B,C, 7A). By contrast, when the muscle driver stock *BG57-GAL4, UAS-GluRIII[RNAi]* is crossed to the same *UAS-Plc21C[RNAi]* transgenic line, full homeostatic compensation occurs (Figures 7B,C). This latter result argues against Plc21C acting in the muscle to control synaptic homeostasis. A prior study demonstrated that presynaptic expression of the same *UAS-Plc21C[RNAi]* construct utilized in our screen (*Plc21C^*GD11359*^*) diminishes *Plc21C* mRNA levels by more than 50% (Dahdal et al., 2010). Moreover, *Plc21C* mRNA is found in the larval nervous system (Shortridge et al., 1991). Collectively, these data are consistent with a neuronal role for Plc21C in the homeostatic control of NMJ function.

To address possible off-target effects of *UAS-Plc21C[RNAi]* expression, we acquired three additional *UAS-Plc21C[RNAi]* lines—Drosophila TRiP constructs (Transgenic RNAi Project) (Ni et al., 2009, 2008). Each TRiP line fails to robustly increase quantal content when crossed to *T15*, (Figure 7A). This result is consistent with an impairment of HSP. Additionally, we examined two heterozygous *Plc21C* deletion mutations: a chromosomal deficiency that removes one copy of *Plc21C* from the genome, *Df(2L)BSC4*, and a 5′ UTR and first exon deletion called *Plc21C^p60A^* (Weinkove et al., 1999). Alone, neither heterozygous deletion diminishes evoked neurotransmission (Figures 7C,D). Heterozygous *Plc21C/+* animals in a *GluRIIA^SP16^* mutant background do have a mild increase in quantal content compared to *Plc21C/+* genetic controls (Figure 7E). However, this increase is not nearly as robust as one would expect for a *GluRIIA* condition (Figure 7E). This result indicates a partial impairment of NMJ homeostatic compensation (Figures 7C,E). Collectively, the data utilizing hypomorphic *Plc21C* conditions support the idea that presynaptic *Plc21C* gates the sustained expression of homeostatic plasticity.

### A role for Gαq in synaptic homeostasis?

**Figure 8:**
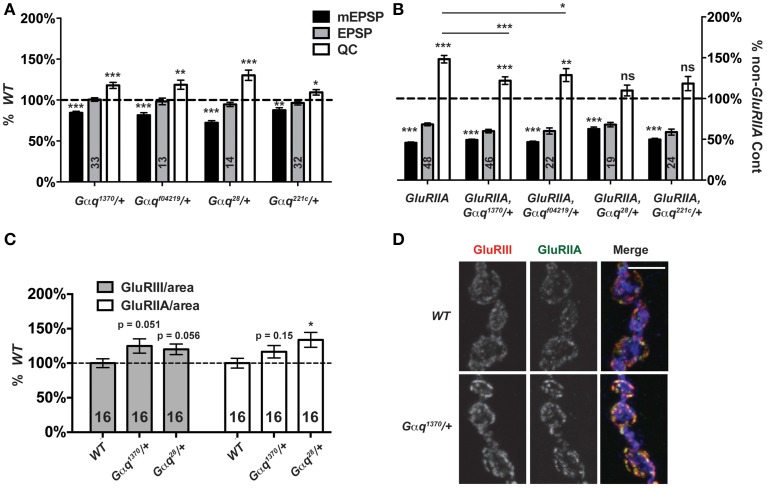
**Partial Gα_q_ loss impairs homeostatic compensation. (A)** Heterozygous loss of *Gαq* does not affect levels of evoked neurotransmission (ns, *p* > 0.05 for each allele compared to *WT*). However, as with loss of *Plc21C*, there is a small decrease in mEPSP amplitude (**p < 0.01 for each allele) and a small increase in QC (*p*-values vary for each allele, but all < 0.05). **(B)** When challenged with a loss of *GluRIIA*, *Gαq^28^*/+ and *Gαq^221c^*/+ NMJs show a complete block in homeostatic compensation (QC—ns, *p* > 0.05) while *Gαq^1370^/+* and *Gαq^f04219^/+* show partial compensation with quantal content elevated slightly compared to genetic control (***p < 0.001 and **p < 0.01, respectively, Student's *T*-Test) but not to the full extent seen in the *GluRIIA^SP16^* control (***p < 0.001 and *p < 0.05). **(C,D)** NMJ glutamate receptor subunit levels per unit of synapse area are not decreased in *Gαq/+* mutants; if anything, they may be slightly enhanced (scale bar, 5 μm). Merged images include anti-GluRIII (red), -GluRIIA (green), and -HRP (blue) staining.

Gαq-GTP and Gβγ G-proteins are classically known to activate PLCβ function (Tedford and Zamponi, 2006). A positive Gαq-Plc21C regulatory relationship appears conserved for Drosophila flight behavior (Banerjee et al., 2006). Therefore, we postulated that *Plc21C* could be playing a role in synaptic homeostasis downstream of G-protein signals. In the course of our screen, we discovered that genetic knock down of a Gαq-encoding gene only mildly impairs synaptic release compared to wild-type: *T15 × UAS-Gαq[RNAi]* (EPSP = 29.8 ± 1.6 mV; *QC* = 64.0 ± 3.7). This effect was not strong (possibly due to partial gene knock down by RNAi) and was not identified as a positive hit from the screen. However, given the results with *Plc21C*, we wished to probe *G*α*q* with genetic mutants. We examined four strong Gαq loss-of-function alleles: *Gαq^1370^*, *Gαq^f04219^*, *Gαq^221c^*, and *Gαq^28^*. Each *Gαq* null allele is homozygous lethal (well before the third instar larval stage), but *Gαq*/+ heterozygotes are viable. For baseline neurotransmission, *Gαq*/+ mutant NMJs show normal levels of postsynaptic excitation (EPSPs, Figure 8A). Interestingly, however, heterozygous *Gαq*/+ NMJs show partially impaired homeostatic plasticity in a *GluRIIA^SP16^* mutant background (Figure 8B).

Each *Gαq*/+ mutant condition shows a small, but significant decrease in NMJ quantal size compared to wild-type controls (Figure 8A). We wondered if this decrease in quantal size could reflect a decrease in glutamate receptor expression levels and a postsynaptic role for Gαq. This possibility could potentially confound our analyses of homeostatic plasticity. Therefore, we stained *Gαq*/+ mutant NMJs with anti-GluRIII and anti-GluRIIA antibodies and quantified stained receptor levels per unit synapse area, as before (Figure 3). We find no significant decrease in glutamate receptor levels in *Gαq*/+ mutant NMJs (Figures 8C,D). In fact, for most measures, there appears to be a slight increase or a statistical trend toward a slight increase of glutamate receptor subunit expression (Figure 8C). This single experiment does not rule out possible postsynaptic functions for Gαq. However, our collective findings are consistent with the possibility that Gαq and Plc21C play concerted roles in the execution of homeostatic plasticity. Further experiments will be required to test if there is a direct regulatory relationship in the presynaptic nerve between the two proteins.

## Discussion

There exists overwhelming evidence that forms of HSP shape how neurons and synapses maintain stable function. Physiologically appropriate levels of synaptic activity must be maintained throughout life while facing numerous endogenous and exogenous challenges to neuronal function. To understand how HSP works on a molecular level, it is important to uncover mechanisms that govern both its initiation and long-term maintenance. Here, we apply prior knowledge and reagents to develop a new genetic stock abbreviated as *T15*: *elaV(C155)-GAL4; Sca-GAL4; BG57-GAL4, UAS-GluRIII[RNAi]*. *T15* allows examination of the long-term maintenance of synaptic homeostasis at the NMJ in a single genetic cross. We validate the synaptic properties of *T15* progeny through a combination of genetics, electrophysiology, and synapse imaging. We demonstrate the utility of *T15* as a genetic tool in multiple ways: (1) we address one aspect of how Ca_V_2 function impinges upon synaptic homeostasis throughout development; (2) we identify CSP as a novel regulator of homeostatic plasticity; and (3) we identify a putative presynaptic system consisting of Drosophila Gαq and PLCβ homologs that also regulates homeostatic plasticity.

### Flexibility and uses of the *T15* drosophila stock

The concept of expressing GAL4-responsive *UAS* transgenes both pre- and post-synaptically at the NMJ is not novel, but it is useful. In our study we add the *UAS-GluRIII[RNAi]* transgene to provide a homeostatic challenge to the NMJ. We have utilized *T15* by crossing it with *UAS-RNAi* transgenes and interrogating the synapse electrophysiologically. Variations on this genetic theme beyond RNAi screening are possible. For example, to test whether NMJ overexpression of a particular *UAS*-driven target gene impairs HSP, a single cross to *T15* would be sufficient. In fact, an “overexpression screen” could be conducted by crossing *T15* to Drosophila stocks carrying transposons with *UAS* insertions in orientations that drive gene expression (Rorth, 1996; Beinert et al., 2004; Bellen et al., 2004; Thibault et al., 2004; Staudt et al., 2005). For another example, viable loss-of-function mutations on the X-chromosome could be directly assayed for roles in homeostatic plasticity. This could be done by crossing mutant females to *BG57-GAL4*, *UAS-GluRIII[RNAi]* males and examining the mutant male larval progeny by electrophysiology. Chromosomal deficiency or duplication screens are also feasible by employing the *BG57-GAL4*, *UAS-GluRIII[RNAi]* chromosome in a similar crossing scheme. With *GluRIIA* genetic mutations, such screens or approaches require more extensive genetic work. The single-cross utility of a *BG57-GAL4*, *UAS-GluRIII[RNAi]* chromosome makes large-scale screens to isolate factors involved in developmental or long-term HSP possible. Finally, *T15* does present hurdles. For example, with *T15*, RNAi-targeted genes are impaired both pre- and post-synaptically. Therefore, the screen itself provides limited information regarding tissue specificity of new homeostatic factors. Follow-up experiments examining positives in the pre- and/or post-synaptic compartments need to be done to garner this information. The advantage of being able to identify both pre- and post-synaptic regulators of HSP in a single-generation crossing scheme outweighs the disadvantage of follow-up work.

### Ca_V_2 channel expression levels and homeostatic synaptic plasticity

Ca_V_2 channels help to evoke fast neurotransmission by allowing an influx of calcium into the presynaptic terminal upon neuronal depolarization (for comprehensive reviews on Ca_V_ channels, see Catterall et al., 2005; Zamponi, 2005). Considerable data from multiple synaptic preparations suggest that Ca_V_2 channels also gate neurotransmitter release in a homeostatic fashion (Frank, 2014b). At the Drosophila NMJ, increased Ca_V_2 function could be directly responsible for a homeostatic potentiation of presynaptic neurotransmitter release (Frank et al., 2006). Yet it is unclear precisely how this process works. It is not known if increased neurotransmission results from trafficking of new Ca_V_2 channels to the synapse, a modulation of functional properties of Ca_V_2 channels already present at the synapse—or even a modulation of Ca^2+^-triggered signaling processes downstream of Ca_V_2 function.

Prior data examining *cac* loss-of-function mutants have not answered this question. Hypomorphic *cac^S^*; *GluRIIA* double mutant NMJs have no significant increase in quantal content compared to *cac^S^* mutant NMJs (Frank et al., 2006). Corroborating this finding, presynaptic Ca^2+^ imaging experiments demonstrate that postsynaptic glutamate receptor impairment correlates with an increase of presynaptic calcium transients (Müller and Davis, 2012)—and *cac^S^* mutant NMJs are particularly resistant to potentiation of function after glutamate receptor impairment (Müller and Davis, 2012). A weaker hypomorphic allele, *cac^TS2^* can impair homeostatic compensation induced by *GluRIIA* deletion, but only if the *cac^TS2^; GluRIIA* animals are reared at a high temperature (29°C)—likely lowering effective *cac* gene function (Frank et al., 2006). Together, these data suggest that absolute amount of Cac/Ca_V_2 function may correlate with the homeostatic capacity of the synapse. Yet additional observations counter this idea. For instance, *cac^S^/+* heterozygous NMJs have nearly normal neurotransmission—significantly better than *cac^TS2^* homozygous NMJs—but *cac^S^*/+; *GluRIIA* NMJs raised at 22°C have partially impaired synaptic homeostasis, while *cac^TS2^; GluRIIA* animals reared at 22°C have intact homeostatic compensation (Frank et al., 2006). Additionally, heterozygous null *cac^HC129^*/+ animals display normal homeostatic compensation (Frank et al., 2009). Taken altogether, it is possible that the presence of dysfunctional Ca_V_2 channel subunits (e.g., Cac^S^) throughout development—rather than the absolute degree of Ca_V_2 dysfunction—could be the driving factor behind impairments of synaptic homeostasis.

Given these background data, it was important to check if severe diminishment of *cac* function is sufficient to impair homeostatic plasticity at the NMJ. Our analyses of *T15 × UAS-cac[RNAi]* NMJs demonstrate that synaptic homeostasis is still intact across multiple concentrations of [Ca^2+^]_e_, even when there is severely defective baseline neurotransmission (Figure 5). It appears if the Ca_V_2 channels present at the synapse are wild-type channels, the NMJ retains its homeostatic capacity—even if the number of functional Ca_V_2 channels that successfully make it to the terminal is sharply reduced. This result is inconsistent with a model in which Ca^2+^-directed signaling downstream of Ca_V_2 activity drives homeostatic plasticity. However, it does not resolve the question of whether the homeostatic potentiation of release normally proceeds through the addition of functional channels to the synapse or through modulation of channel properties. According to a “slot model” of Ca_V_2.1 channel positioning, at presynaptic terminals there may exist only a limited number of channel-type-specific slots (Cao et al., 2004). If this were true at the Drosophila NMJ—and if all slots were normally occupied—it would point to modulation of Ca_V_2 gating properties as a probable mechanism.

A recent paper corroborates this idea. Mutations in two Drosophila genes encoding epithelial sodium (ENaC) channels block both the rapid induction and long-term expression of synaptic homeostasis—importantly, by impairing necessary increases in presynaptic calcium influx (Younger et al., 2013). Interestingly, when the ENaC antagonist benzamil is acutely applied to *GluRIIA* mutant NMJs, presynaptic calcium influx is dramatically decreased. By contrast, benzamil has no effect on presynaptic calcium influx at wild-type NMJs (Younger et al., 2013). Collectively, these observations are consistent with a model in which Ca_V_2 gating properties are enhanced during synaptic homeostasis, with ENaC channels mediating this enhancement (Younger et al., 2013).

### CSP and a sustained expression of homeostatic plasticity

CSP was originally identified in Drosophila from an antibody-based approach as a synapse-specific antigen (Zinsmaier et al., 1990). Follow-up work has demonstrated that CSP executes multiple synaptic functions (Zinsmaier, 2010; Donnelier and Braun, 2014). Interestingly, loss of CSP function appears to exacerbate synaptic problems over developmental time in organisms as varied as Drosophila (Zinsmaier et al., 1994), mice (Fernandez-Chacon et al., 2004; Garcia-Junco-Clemente et al., 2010), and humans (Benitez et al., 2011; Noskova et al., 2011; Velinov et al., 2012). These facts could be in line with data that CSP is required for the long-term maintenance of homeostatic plasticity—even though CSP is not required for its short-term induction (Dickman and Davis, 2009) (Figure 6F).

What is the specific role of CSP during the execution of HSP? This is unclear. Based on the literature, there are several plausible connections. As stated above, modulation of Ca_V_2 functional properties could be key to synaptic homeostasis, and CSP has previously been implicated in the modulation of Ca_V_2-type calcium channel function (Gundersen and Umbach, 1992). We observe a genetic interaction between *Csp* and *cac* loss-of-function mutations (Figure 6G)—however, other studies have failed to find evidence for direct Ca_V_ regulation by CSP, including studies at Drosophila synapses (Morales et al., 1999; Dawson-Scully et al., 2000). Therefore, other modes of regulation that genetically interact with *cac* are likely. For example, CSP has been implicated in the regulation of Ca^2+^-triggered exocytosis (Dawson-Scully et al., 2000; Bronk et al., 2001; Dawson-Scully et al., 2007), and recent studies have investigated possible CSP chaperone interactions with SNARE molecules. SNAP25 (t-SNARE) expression is significantly decreased in mice lacking CSPα (Chandra et al., 2005; Zhang et al., 2012). This represents a possible link to homeostatic plasticity because reduction of SNAP25 levels compromises homeostatic signaling at the Drosophila NMJ (Dickman et al., 2012).

### Gαq, PLCβ, and lipid signals

Our data demonstrate that partial losses of *Gαq* and *Plc21C* gene function partially impair the homeostatic response (Figures 7, 8). The data open several possibilities for presynaptic control of neurotransmitter release and homeostatic plasticity. By one model, diminishment of PLCβ signaling could result in excess PIP_2_ at the synapse at the expense of IP_3_ and membrane-bound DAG. In other systems, it is well known that DAG activates protein kinase C (PKC), which in turn, activates Ca_V_2 channels (Tedford and Zamponi, 2006). In this way, DAG-mediated regulation of Ca_V_2 function could be a key determinant in homeostatic plasticity. However, by a second model, it has been reported that *Plc21C* gene function and DAG regulate the abundance of synaptic DUNC-13 at the Drosophila NMJ (Aravamudan and Broadie, 2003). DUNC-13 is a highly conserved protein critical for proper maintenance of SNARE-mediated vesicle exocytosis (Jahn and Fasshauer, 2012; Kasai et al., 2012; James and Martin, 2013), a process previously implicated in homeostatic plasticity at the NMJ (Dickman et al., 2012). There exists evidence that DUNC-13 family molecules could regulate the dynamics of the readily releasable pools (RRP) of synaptic vesicles (Chen et al., 2013). RRP size enhancement is another process implemented during synaptic homeostasis at the NMJ (Weyhersmüller et al., 2011; Müller et al., 2012). For a third model, depressed Gαq/PLCβ signaling could result in lower levels of synaptic IP_3_. Canonically, intracellular IP_3_ binds to IP_3_ receptors and liberates Ca^2+^ from intracellular stores. In turn, Ca^2+^-dependent signaling cascades could permit the expression of homeostatic plasticity. Finally, PIP_2_ itself is known to bimodally regulate Ca_V_2 channels: Ca_V_2 channels are stabilized by low levels of PIP_2_, but inhibited by high levels of PIP_2_ (Wu et al., 2002). Interestingly, an amino-acid substitution on the intracellular side of transmembrane domain III S6 on Ca_V_2.1 alters its affinity for PIP_2_. This is the same domain containing the amino-acid substitution in *cac^S^* (Smith et al., 1998; Zhen et al., 2006). More experiments are required to define a precise mechanism of Gαq/PLCβ signaling in the execution of synaptic homeostasis at the NMJ. Regardless of mechanism, the results implicating Gαq and Plc21C in synaptic homeostasis suggest that a synaptic signal controls presynaptic excitability through a G-Protein Coupled Receptor (GPCR)/Gαq/PLCβ-mediated signaling pathway. This is an exciting possibility that is open to exploration via additional genetic approaches in Drosophila, taking advantage of tools like those we generated for this study.

### Conflict of interest statement

The authors declare that the research was conducted in the absence of any commercial or financial relationships that could be construed as a potential conflict of interest.
