# Supplementary material for: A single-cross, RNA interference-based genetic tool for examining the long-term maintenance of homeostatic plasticity
Source: Front Cell Neurosci. 2015 Mar 26;9:107. doi: 10.3389/fncel.2015.00107 (PMC4374470; doi:10.3389/fncel.2015.00107)
Supplement: Supplementary file 1 [file Table1.PDF]

Genotype	n	mEPSP (mV)	mFreq (Hz)	EPSP (mV)	QC (T-test vs control)	NLS QC (T-test vs control)	RMP (mV)	Figure
WT	31	0.81 ± 0.04	4.18 ± 0.26	34.2 ± 0.6	43.8 ± 2.1	82.8 ± 4.7	-62.3 ± 0.5	1, 2
BG57/UAS-GluRIII[RNAi]	6	0.46 ± 0.05	0.83 ± 0.14	33.6 ± 2.7	**76.6 ± 7.1** **	**139.4 ± 16.0** **	-66.1 ± 1.4	1
elaV(C155); Sca; BG57 x WT (GAL4 cont x WT)	34	0.90 ± 0.05	2.05 ± 0.15	33.6 ± 1.0	42.0 ± 2.1	81.2 ± 4.8	-65.8 ± 0.7	2
T15 x WT	30	0.49 ± 0.02	0.71 ± 0.12	32.6 ± 0.8	**68.6 ± 2.4** ***	**123.6 ± 5.8** ***	-64.6 ± 0.7	2
GAL4 cont x UAS-cac[RNAi] (0.5 mM [Ca^{2+}])	10	0.71 ± 0.04	2.91 ± 0.57	5.6 ± 0.7	8.1 ± 1.2	9.0 ± 1.4	-61.9 ± 1.7	5
T15 x UAS-cac[RNAi] (0.5)	11	0.35 ± 0.03	0.64 ± 0.10	5.5 ± 1.2	**16.0 ± 3.3** *	**18.1 ± 4.1** #	-61.3 ± 1.1	5
GAL4 cont x UAS-cac[RNAi] (1.5)	8	0.68 ± 0.04	2.75 ± 0.09	26.6 ± 1.4	39.1 ± 1.5	61.9 ± 3.0	-62.7 ± 1.6	5
T15 x UAS-cac[RNAi] (1.5)	8	0.28 ± 0.02	0.59 ± 0.08	21.3 ± 1.0	**77.0 ± 4.7** ***	**109.6 ± 7.2** ***	-62.1 ± 0.8	5
GAL4 cont x Csp[RNAi]	8	0.79 ± 0.04	1.84 ± 0.38	30.3 ± 1.7	39.3 ± 3.5	68.1 ± 7.7	-63.9 ± 0.6	6
T15 x Csp[RNAi]	5	0.49 ± 0.04	0.36 ± 0.05	23.4 ± 1.0	**49.0 ± 4.9** ns	**73.6 ± 8.6** ns	-61.1 ± 0.6	6
$Csp^{DG29203}$	11	0.87 ± 0.06	4.64 ± 0.39	27.1 ± 1.0	32.8 ± 2.4	53.6 ± 4.5	-61.0 ± 0.2	6
$GluRIIA^{SP16}$; $Csp^{DG29203}$	7	0.32 ± 0.02	0.76 ± 0.11	11.7 ± 1.3	**36.5 ± 4.0** ns	**44.2 ± 5.3** ns	-61.8 ± 0.8	6
$Csp^{EY22488}$/+	19	0.64 ± 0.02	3.21 ± 0.33	31.9 ± 1.3	50.5 ± 2.2	90.4 ± 5.3	-64.5 ± 0.6	6
$GluRIIA^{SP16}$; $Csp^{EY22488}$/+	13	0.33 ± 0.01	0.45 ± 0.07	14.6 ± 1.0	**44.5 ± 3.2** ns	**56.8 ± 4.8** ***lower	-61.4 ± 0.4	6
GAL4 cont x WT (new)	15	0.83 ± 0.06	1.86 ± 0.20	37.9 ± 1.2	48.5 ± 3.4	97.9 ± 7.5	-66.0 ± 0.9	7
T15 x WT (new)	23	0.53 ± 0.02	0.70 ± 0.04	32.6 ± 1.1	**70.2 ± 2.7** ***	**139.1 ± 7.2** ***	-65.8 ± 1.1	7
T15 x $Plc21C^{GD11359}$[RNAi]	14	0.51 ± 0.02	0.39 ± 0.07	25.7 ± 1.7	**50.8 ± 3.4** ns	**79.1 ± 7.5** ns	-67.3 ± 1.4	7
Df(2L)BSC4/+	17	0.67 ± 0.02	4.80 ± 0.32	33.1 ± 1.0	50.7 ± 2.4	93.4 ± 6.4	-64.2 ± 0.9	7
Df(2L)BSC4, GluRIIA/+, GluRIIA	34	0.38 ± 0.01	2.25 ± 0.26	21.9 ± 1.1	**59.2 ± 3.2** **	**88.2 ± 6.2** ns	-63.6 ± 0.6	7
$Plc21C^{p60A}$/+	15	0.68 ± 0.04	4.28 ± 0.36	35.2 ± 0.8	53.2 ± 2.7	99.8 ± 6.2	-66.7 ± 0.7	7
$Plc21C^{p60A}$, GluRIIA/+, GluRIIA	10	0.33 ± 0.02	1.59 ± 0.14	21.6 ± 1.6	**67.3 ± 5.4** *	**97.1 ± 10.0** ns	-63.8 ± 1.1	7
$G\alpha q^{1370}$/+	33	0.68 ± 0.02	4.14 ± 0.32	34.1 ± 1.0	51.3 ± 2.1	97.5 ± 5.1	-63.5 ± 0.7	8
GluRIIA, $G\alpha q^{1370}$/GluRIIA, +	46	0.34 ± 0.01	1.33 ± 0.09	20.6 ± 0.7	**61.4 ± 2.4** ***	**88.6 ± 4.3** ns	-61.8 ± 0.3	8
$G\alpha q^{28}$/+	14	0.59 ± 0.02	3.90 ± 0.32	32.2 ± 0.9	55.6 ± 2.7	100.4 ± 6.1	-63.1 ± 0.9	8
GluRIIA, $G\alpha q^{28}$/GluRIIA, +	19	0.37 ± 0.1	1.40 ± 0.14	21.9 ± 0.9	**61.1 ± 3.7** ns	**89.1 ± 6.3** ns	-62.6 ± 0.8	8

Supplementary Table 1: Selected raw electrophysiological data. Values are mean ± SEM. n (number of NMJs recorded for the indicated genotype); mEPSP (average miniature excitatory postsynaptic potential); EPSP (average excitatory postsynaptic potential); QC (average quantal content); NLS QC (average QC corrected for non-linear summation); RMP (average resting membrane potential). Similar genotypes are excluded for the sake of space. T-test comparisons to control (usually in row directly above) are provided for QC and NLS QC for assessment of homeostatic increases in presynaptic release. Specific *p* values or *p* value ranges match those in the text, figures, and figure legends, with $p < 0.05$ marked as significant ($^{\#}$ $p = 0.05$; * $p < 0.05$; ** $p < 0.01$; *** $p < 0.001$). Data sets marked as "new" are repeats of an identical genotype earlier in the table. In those cases, new control data was collected for a new experiment to make ensure control and experimental subjects had matching recording conditions (saline and electrophysiologist). Only NMJ recordings with an input resistance of > 5 MΩ and a sufficiently low RMP (< -56 mV) were accepted for analysis. In no case above do average RMPs between experimental and control data sets differ by more than 4.0 mV.
